# Analysis of the Proteome and Biochemistry of Venom from *Tityus confluens*, a Scorpion That Can Be Involved in Severe Envenomation Cases in Brazil

**DOI:** 10.3390/toxins17080406

**Published:** 2025-08-14

**Authors:** Laís Corrêa Lima, Henrique Ranieri Covali-Pontes, Ohanna Gabriely Souza Leite, Renata Trentin Perdomo, Luiz Filipe Ramalho Nunes de Moraes, Ludovico Migliolo, Mauricio Nogueira Moyses, Natália Gabrielly Pereira dos Santos, Daniel Carvalho Pimenta, Mariana Soares Rodrigues, Karen Morais-Zani, Guilherme Rabelo Coelho, Malson Neilson Lucena

**Affiliations:** 1 Institute of Biosciences, Federal University of Mato Grosso do Sul, Campo Grande 79070-900, MS, Brazil; 2 Faculty of Pharmaceutical Sciences, Food and Nutrition, Federal University of Mato Grosso do Sul, Campo Grande 79070-900, MS, Brazil; 3 S-Inova Biotech, Postgraduate Program in Biotechnology, Dom Bosco Catholic University, Campo Grande 79117-900, MS, Brazil; 4 Laboratório de Bioquímica, Butantan Institute, São Paulo 05503-900, SP, Brazil; 5 Laboratório de Ecologia e Evolução, Butantan Institute, São Paulo 05503-900, SP, Brazil; 6 Programa de Tecnologia Nuclear – Aplicações, Instituto de Pesquisas Energéticas e Nucleares, São Paulo 05508-000, SP, Brazil; 7 Laboratório de Fisiopatologia, Butantan Institute, São Paulo 05503-900, SP, Brazil

**Keywords:** scorpion venom, enzymes, envenomation, hypotension, K^+^ channel toxin, Na^+^ channel toxin

## Abstract

In Brazil, the annual scorpion sting cases surpass those of other neglected tropical diseases, highlighting a significant public health issue. The severity of scorpion envenomation relates to the venom’s rapid action, complex composition, species identification challenges, and limited antivenom availability. This work aimed to characterize the venom of *Tityus confluens* through proteomic, enzymatic, and biological analyses while also assessing its reactivity to anti-scorpion antivenom. The electrophoretic analysis revealed seven protein bands, with the most prominent bands at 30, 15, and 10 kDa. The C18-RP-HPLC analysis isolated sixteen primary fractions. The proteomic analysis identified various toxins, including potassium channel toxins, sodium channel toxins, and antimicrobial peptides, as well as other proteins such as hypotensin and metalloproteinases. Antigenic components were identified in the *T*. *confluens* venom, which displayed dose-dependent but time-independent amylolytic activity. The ATPase activity significantly increased with 1–10 μg of venom. No cytotoxic effects were observed on carcinoma or non-tumoral cell lines. The *T*. *confluens* venom features a complex protein composition rich in toxins that target ion channels and enzymes. It exhibits active enzymatic and antigenic properties, and displays low cytotoxicity. This is the first proteomic research on the composition of *T*. *confluens* venom and may provide valuable insights into understanding the clinical manifestations of scorpion stings.

## 1. Introduction

Since 2017, Brazil has recorded 120,000 annual scorpion stings [1], exceeding the incidence of the country’s most neglected tropical diseases [2]. The state surveillance system in Mato Grosso do Sul shows a concerning upward trend, increasing from 1064 cases in 2012 to 4922 cases in 2024, with a lethality rate of 24 confirmed deaths during this period [1]. Between 2014 and 2023, Brazil reported 1,171,846 cases [3]. Therefore, scorpion envenomation poses a serious public health issue.

Given the potency and rapid action of the venom, immediate medical attention is crucial, often requiring the administration of antivenom and intensive care [3]. The lethality of scorpion envenomation is linked to the swift distribution of toxins in the body, delays in the proper administration of treatment caused by difficulties in identifying the scorpion species or the unavailability of antivenom, and the variety of compounds in scorpion venom [2,3].

Scorpion venoms contain peptides known as neurotoxins that target sodium, potassium, chloride, and calcium channels, along with other peptides that have cytotoxic, antimicrobial, anticancer, pro-inflammatory, antifungal, and antiparasitic properties. Additionally, scorpion venoms include enzymes such as phospholipases, metalloproteases, and hyaluronidases [4,5,6].

Buthidae comprises 94 genera and 1284 described species distributed worldwide, except for Antarctica and New Zealand [7]. About 95% of human scorpion envenomation cases are caused by scorpions from the Buthidae family, with the main genera being *Tityus*, *Centauroids*, and Butkus [8]. *Tityus* includes around 220 species found across Central America and South America, from Costa Rica to Argentina [9,10]. Mato Grosso do Sul has recorded 16 species and one non-nominotypical subspecies [11].

*Tityus confluens* was initially described as a subspecies of *Tityus trivittatus* and was later elevated to species status in a more detailed research. This separation was corroborated by morphological characteristics that distinguish *T*. *confluens* from other subspecies of *T*. *trivittatus* [12]. *T*. *confluens* is part of the scorpion fauna of the Chaco region and is now extensively synanthropic, found in Argentina, Brazil, Bolivia, and Paraguay [13,14]. In Brazil, it is present in the states of Mato Grosso, Mato Grosso do Sul, Goiás, Tocantins, Rondônia, Ceará, Piauí, Santa Catarina, and Paraná [9,11,15].

*Tityus confluens* appears to be a parthenogenetic species, at least in some areas of its known distribution range [14]. The toxicity of the venom, including the median lethal dose (LD50), course of experimental envenomation, histopathological lesions, and immunochemical characteristics, has been characterized for the venom of *T*. *confluens* from Argentina [16]. Recently, *T*. *confluens* venom has been demonstrated to increase extracellular ATP levels and decrease extracellular adenosine (ADO) levels. Additionally, this research revealed the inherent ATPase activity of *T*. *confluens* venom and its ability to influence the activities of E-NTPDase, E-5′-NT, and E-ADA in rat blood cells [17].

Despite this, the composition of *T*. *confluens* venom remains unknown. Reports of scorpion envenomation from this species have increased significantly in recent years, particularly in Brazil’s Midwest region [2]. Furthermore, this species has already been associated with human fatalities in Argentina. In light of this situation, *T*. *confluens* ought to be regarded as a potential public health threat in Brazil. This work aimed to characterize *T*. *confluens* venom using two different approaches—through venom proteomics and enzymatically or biologically. In addition, the immunological reactivity of the venom was tested against anti-scorpion antivenom. Our research marks the first proteomic investigation into the composition of *T*. *confluens* venom. The proteomic analysis revealed the presence of sodium channel toxins, potassium channel toxins, antimicrobial peptides, hypotensin, and metalloproteinases.

## 2. Results

### 2.1. Electrophoretic Profile

**Figure 1:**
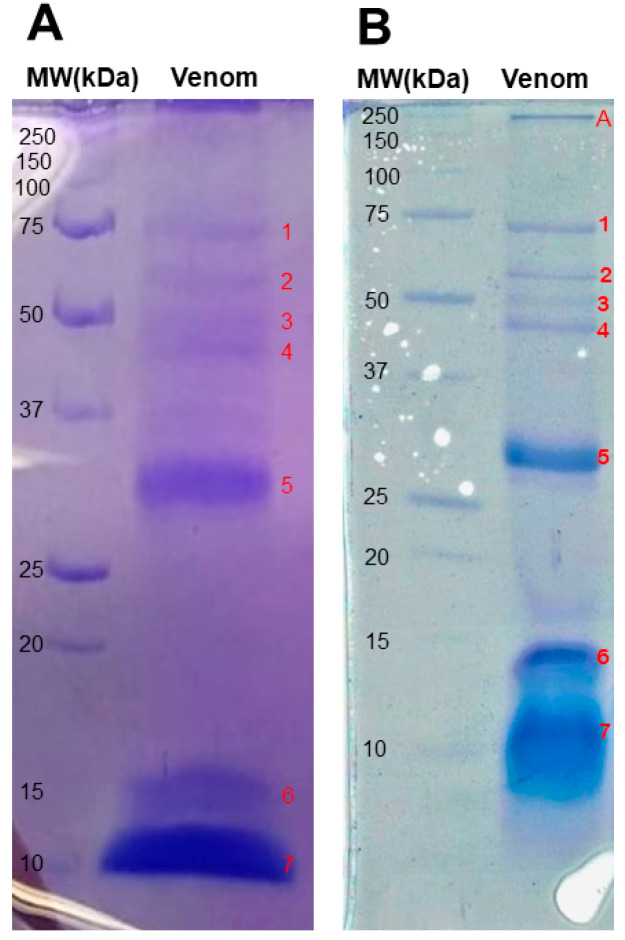
Electrophoretic profile of venom and hemolymph of *Tityus confluens* (25 µg of protein) following Coomassie Blue staining: (**A**) glycine SDS-PAGE; (**B**) tricine SDS-PAGE. Lane 1, molecular weight marker; lane 2, venom; lane 3, molecular weight marker; lane 4, venom. The numbers indicate that the same band can be observed in both gels. The letters denote new bands in tricine SDS-PAGE.

The analysis of *T*. *confluens* venom proteins, assessed using 15% glycine SDS-PAGE, revealed the presence of seven molecular weight bands at 75, 60, 50, 45, 30, 15, and 10 kDa (Figure 1A). The major bands were detected at 30, 15, and 10 kDa. The analysis using tricine SDS-PAGE showed two new bands at 250 and 18 kDa (Figure 1B).

### 2.2. HPLC Analysis

**Figure 2:**
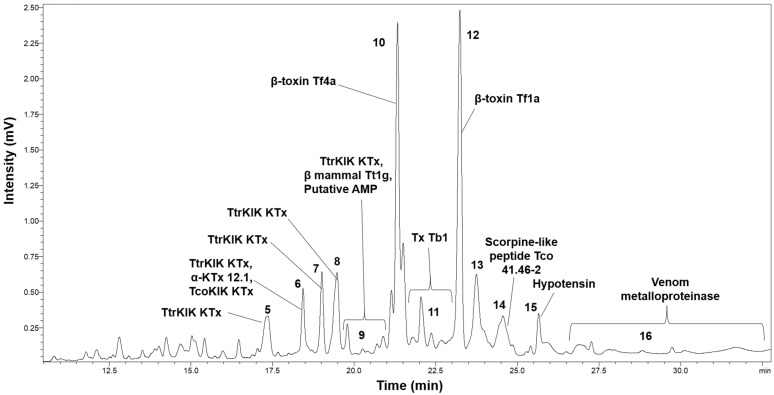
Annotated representative profile of *Tityus confluens* venom using C18-RP-HPLC. Fractions are sequentially numbered based on the separation strategy, and the peak labels present the UniProt accession code for the proteomically identified major toxins found in the chromatographic fractions.

The chromatographic profile from the C18-RP-HPLC venom fractionation during *Tityus confluens* venom decomplexation is presented in Figure 2, which was annotated based on subsequent proteomic identification. The HPLC analysis revealed approximately sixteen distinct peaks. A total of eleven peaks were identified, as shown in Figure 2. The venom profile demonstrated that most peaks were eluted between retention times of 12.5 and 27.5 min, with no additional fractions observed beyond 32 min.

### 2.3. Proteomic Analysis

**Figure 3:**
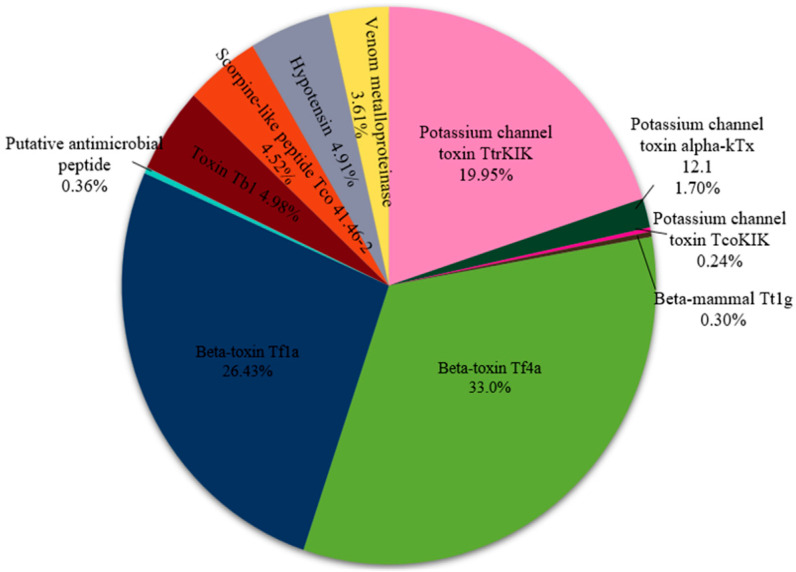
Relative distribution of toxins in *T*. *confluens* venom, evaluated by protein quantity. We assessed the quantities based on the corresponding chromatographic peak areas of the identified toxin(s).

After fractionation and identification, the venom proteins and peptides were thoroughly analyzed, as summarized in Table 1 and Figure 3. Sixteen toxins were identified in the venom of *Tityus confluens*, including potassium channel toxins (TtrKIK, scorpion-like peptides Tco 41.46-2 and alpha-KTx 12.1), sodium channel toxins (β-mammal Tt1g, β-toxin Tf4a, Toxin Tb1, and β-toxin Tf1a), antimicrobial peptides (putative antimicrobial and scorpion-like peptide Tco 41.46-2), hypotensin, and venom metalloproteinases (Table 1). Sodium and potassium channel toxins are the most common molecules, accounting for 61% and 22%, respectively. hypotensin, metalloproteinases, and antimicrobial peptides are also present but in lower amounts (Figure 3).

### 2.4. Western Blotting Analysis

**Figure 4:**
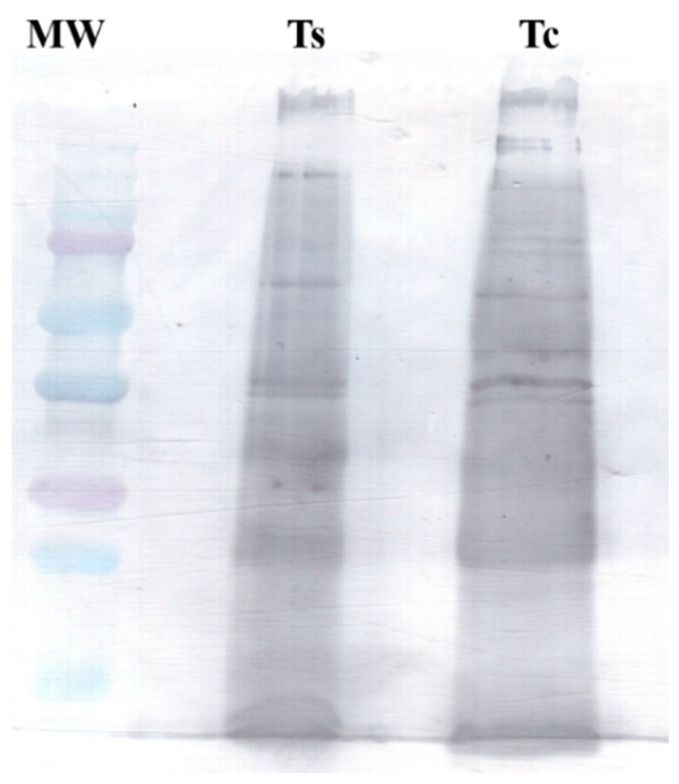
Antigenic profile of the venom from *T. confluens.* The membrane was incubated with a 1:1000 dilution of anti-scorpion serum (batch 190215) for 2 h. Lane 1: molecular weight marker; lane 2: Ts—venom from *T. serrulatus*; lane 3: Tc—venom from *T. confluens*.

Using Western blotting, we identified several antigenic components in the venom of *T. confluens* (Figure 4). The antigenic profile observed for the *T. confluens* venom was similar to that of the *Tityus serrulatus* venom.

### 2.5. Amylolytic Activity

**Figure 5:**
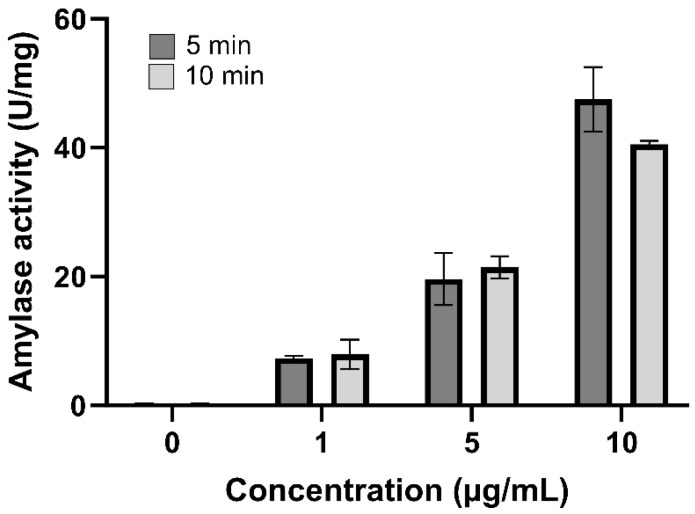
The amylolytic activity of crude venom from *T. confluens* was examined. A negative control (0.02 M sodium phosphate buffer, without venom) and three venom concentrations (1, 5, and 10 μg) were utilized. Results are presented as the mean (*n* = 3) ± standard deviation (SD).

The presence of amylase in the *T. confluens* venom was assessed by measuring the amylolytic activity using the DNS method (Figure 5). All concentrations of venom displayed amylolytic activity, significantly increasing the activity to 50 U/mg. While the amylolytic activity is dose-dependent, it is not time-dependent, as there are no differences in activity between 5 and 10 min.

### 2.6. Effect of Venom on Total ATPase Activity

**Figure 6:**
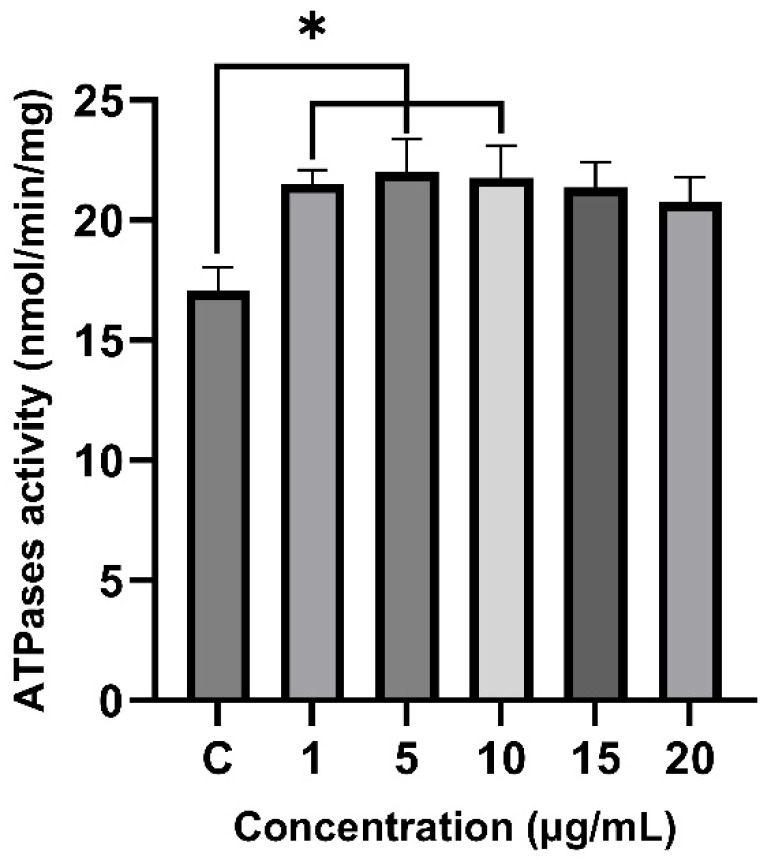
Effect of the venom from *T. confluens* on renal total ATPase activity. Results are presented as the mean ± standard deviation of three independent assays. Data were analyzed using a one-way analysis of variance followed by Tukey’s honestly significant difference test; * indicates *p* < 0.05 compared to control.

The effect of the venom from *T. confluens* on the total renal ATPase activity was assessed with various concentrations of the venom (Figure 6). After 30 min of incubation, the total ATPase activity increased to 26%, 29%, and 27% with 1 μg (*p* = 0.04597), 5 μg (*p* = 0.02841), and 10 μg (*p* = 0.03558) of venom, respectively. No significant increase in activity was observed with 15 or 20 μg of venom.

### 2.7. Cytotoxicity

**Figure 7:**
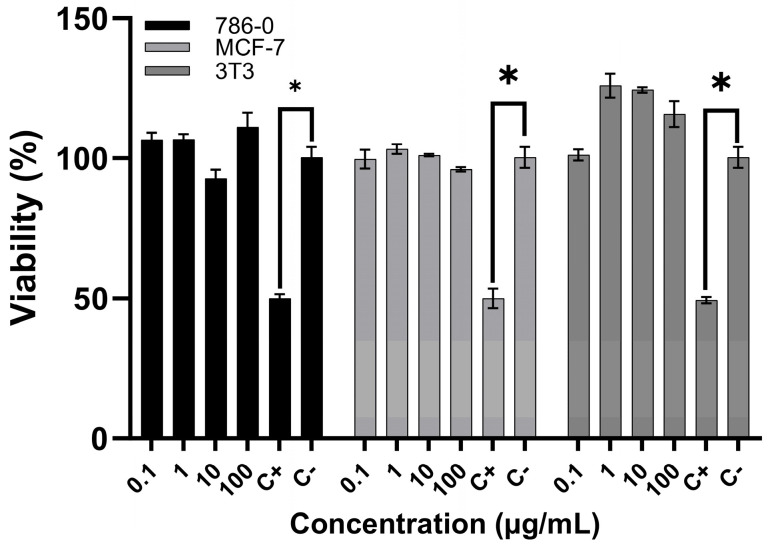
Effects of venom of *T. confluens* on the viability of carcinoma cells and non-tumoral fibroblasts. Cells were treated with different concentrations of the venom for 48 h. Cell viability was determined via the sulforhodamine B assay. Results are the mean ± standard deviation of three independent experiments conducted in triplicate. C^+^, positive control, doxorubicin; C^−^, negative control, without venom. * indicates *p* < 0.05 compared to positive control.

The viability of three cell lines—renal carcinoma, invasive breast carcinoma, and non-tumoral fibroblasts—was not affected by exposure to *T. confluens* venom at concentrations up to 100 μg/mL (Figure 7).

## 3. Discussion

The electrophoretic profile of the *T. confluens* venom displayed similarities to protein bands previously documented for other scorpion species [5]. The electrophoresis results consistently showed three bands (2, 3, and 4) with molecular weights ranging from 55 to 45 kDa. Previous research on *T. serrulatus* venom identified a 51 kDa purified hyaluronidase [18]. Band 1 (~70 kDa) matches the molecular weight of hemocyanins, which are abundant in arthropod hemolymph and present in scorpion venom [19]. The diffuse band and migration below 10 kDa indicate the presence of low-molecular-weight proteins, which are common in scorpion venoms. For instance, *Mesobuthus tamulus* contains ion-channel-binding toxins (Na^+^ and K^+^) ranging from 3 to 15 kDa [20]; *T. serrulatus* has Na^+^ channel toxins (NaScTxs) within the range of 6500 to 8500 Da [21]. These low-molecular-weight bands support the presence of peptides, typically 3.0 to 5.0 kDa or 6.0 to 8.0 kDa in mass, which are crucial to venom toxicity [22].

Mass-spectrometry-based proteomics allows for protein quantification using label-free or label-based methods. Label-free quantification (LFQ) is cost-effective and widely applicable but has limitations related to missing values, ion suppression, and technical variability [23]. Label-based approaches, including SILAC and TMT, offer higher accuracy and enable the multiplexing of samples, although they are more expensive and susceptible to issues such as ratio compression in isobaric labeling [24]. A major challenge with all mass-spectrometry-based strategies is the limited dynamic range of detection compared to the wide range of protein abundance in biological samples [25]. In venom proteomics, small neurotoxins often dominate the spectra, while lower-abundance enzymes may be underrepresented due to ionization biases and the complexity of the venom matrix [26].

Animal venoms typically consist of various neurotoxins designed to disrupt the nervous systems of predators or prey. Scorpion venoms are rich in peptide toxins, which modulate voltage-gated sodium channels essential for initiating and propagating action potentials in excitable cells. These toxins are primarily classified into two types, α-toxins and β-toxins, each targeting distinct sites on the sodium channels and altering their gating properties [27]. Beta toxins bind to site 4 of sodium channels, leading to a shift in their activation potential, making them more likely to open at more negative membrane potentials. The sodium-channel-modulating toxins found in the venom of *Tityus confluens* belong to a family of β-mammal toxins that are widely distributed across the Tityus genus. This includes toxins such as Ts1 (*T. serrulatus*), Tb1 (*T. bahiensis*), Tz1 (*T. zulianus*), Tt1g (*T. trivittatus*), Tf4a (*T. fasciolatus*), and Tst1 (*T. stigmurus*), all of which exhibit similar activity on neuronal sodium channels [28,29].

The β-mammal toxins that modulate sodium channels bind to the voltage sensor in domain II of these channels, facilitating early activation and enhancing neuronal excitability [30]. This may result in hyperexcitability, muscle spasms, involuntary contractions, intense pain, and autonomic dysfunctions such as tachycardia and sweating. In severe cases, it can lead to cardiac arrhythmias [28,31,32]. Therefore, these physiological manifestations can also be attributed to *T. confluens* envenomation.

Scorpion venom toxins, which act as ligands for K^+^ channels (KTx), consist of polypeptides containing 23 to 64 amino acid residues [33]. α-KTx 12.1 functionally reversibly inhibits several potassium channels, including voltage-gated channels Kv1.2 and Kv1.3, as well as calcium-activated channels KCa1.1 and KCa3.1. This peptide was isolated from the venoms of four *Tityus* scorpion species—*T. serrulatus*, *T. bahiensis*, *T. stigmurus*, and *T. trivittatus* [34,35,36,37,38].

The toxin Tco 41.46-2 is a scorpine-like peptide isolated from the venom of the Brazilian scorpion *Tityus costatus* [39]. This conformation allows the toxin to interact with specific subtypes of potassium channels, demonstrating a hierarchical binding affinity of Kv1.2 > Kv1.3 > Kv1.1 > Kv1.6 [39]. The research indicates that Tco 41.46-2 may reduce the replication of the parasite *Toxoplasma gondii* by stimulating protective inflammatory responses in host cells rather than exerting a direct parasiticidal effect [40].

Scorpion venom contains a variety of bioactive peptides, notably a group known as hypotensins, which are recognized for their blood-pressure-lowering effects. These peptides have attracted attention for their potential therapeutic uses in cardiovascular diseases. A specific example is the hypotensin from *T. serrulatus* (TsHpt), which are linear peptides found in the venom of *T. serrulatus*. The first characterized member, TsHpt-I, enhances the effects of bradykinin, a vasodilator, resulting in reduced blood pressure in normotensive rats [41]. In addition to TsHpt, other components in scorpion venom have been shown to have hypotensive effects. For example, the phospholipase A_2_ enzymes present in some scorpion venoms can induce a rapid, temporary decrease in blood pressure, followed by a hypertensive response caused by neurotoxic polypeptides [42]. Furthermore, a tripeptide found in scorpion venom, Lys-Pro-Pro (KPP), has demonstrated positive effects on the cardiovascular system of hypertensive rats, suggesting its potential for therapeutic development [43].

Although they are more commonly found in snake venoms, metalloproteinases have also been identified in scorpion venoms, including those from the species *Tityus serrulatus* and *Tityus discrepans* [44,45]. These enzymes contribute to the proteolytic activity of the venom by facilitating the degradation of extracellular matrix components, which can enhance the diffusion of other toxins and modulate inflammatory responses [46]. Furthermore, metalloproteinases can activate protoxins through post-translational modifications, inhibit platelet aggregation, modulate cytokine production, and activate the complement system, thereby amplifying the toxic effects of the venom [46].

Although only metalloproteinases have been identified in *T. confluens*, studies of scorpions from the *Tityus* genus using proteomic and transcriptomic analyses have shown that their venoms contain several key enzymes that contribute to their complexity and toxicity. Among the most frequently identified enzymes are hyaluronidases, which assist in venom dispersal by breaking down extracellular matrix components and have been studied in species such as *T. serrulatus* [47]. Although phospholipase activity is less common in scorpion venoms, functional phospholipase-like enzymes have been found in *T. stigmurus* venom through proteomic and transcriptomic methods [48]. Additionally, serine proteases that may be involved in proteolytic degradation have been discovered in the venoms of *T. obscurus* and *T. serrulatus* [49]. Finally, transcriptomic data have revealed the presence of L-amino acid oxidases (LAAOs), enzymes linked to antimicrobial and pro-inflammatory effects, in *T. obscurus* [50]. Together, these enzymatic components demonstrate the diverse functional roles of Tityus venoms, providing insights into their pathophysiological effects.

Scorpion antivenom effectively neutralizes toxins, reduces symptom severity, and safeguards health and life. Scorpion antivenom is produced by immunizing horses with a blend of venoms from different scorpion species [51]. In Brazil, this includes 50% venom from *T. serrulatus* and 50% from *T. bahiensis* [52]. In this work, we identified various antigenic components present in the venom of *T. confluens* using scorpion antivenom. This suggests immunochemical cross-reactivity between the venoms of *T. confluens* and *T. serrulatus*, aligning with the cross-reactivity noted among *Tityus* venoms [16,53]. It has been proposed that a monovalent antivenom might help neutralize the venom from Brazilian scorpions [53]. After a scorpion sting, it is advisable to treat the wound with species-specific antivenom. If that is not available, the antivenom for *T. serrulatus* should be administered in cases of *T. confluens* stings. Considering the serious consequences of *T. confluens* envenomation, it is crucial to conduct specific preclinical studies, including ELISA, antivenomics, and in vivo assays. These studies will help validate treatment protocols and develop more effective or region-specific antivenoms.

Although the role of amylases in venom remains unclear, proteomic analyses have identified alpha-amylases in *Centruroides limpidus* and similar enzymes in *T. serrulatus* and *T. obscurus* [54]. In the venom of *Rhopalurus agamenon*, the presence of amylase was confirmed through colorimetric, zymographic, and proteomic methods, making it the second most abundant enzyme (23%) [55]. While some scorpion stings are known to raise serum amylase levels due to pancreatic damage, it is still unclear whether this enzyme is present in the venom linked to this symptom [55]. Even though the venom showed enzymatic activity, our proteomic analysis did not identify any matching enzymes. This suggests that the venom contains low-abundance components or peptides with enzymatic functions that are not in known protein databases.

ATPases are membrane-bound enzymes that hydrolyze the terminal phosphate of ATP. Along with degradation products such as ADP, AMP, and adenosine, they participate in biological processes including neurotransmission, muscle contraction, cancer, pain, and inflammation. Na/K-ATPase serves as the primary ion-transporting protein in eukaryotic cells. Animal toxins can modulate its activity; for example, melittin (from *Apis mellifera* venom) inhibits Na/K-ATPase [56]. Arenobufagin from *Peltophryne fustiger* also inhibits this enzyme, similar to other bufadienolides [57,58]. Conversely, the *Bothrops alternatus* venom increases renal Na/K-ATPase activity levels [59]. This increase in Na/K-ATPase activity is a crucial component of the body’s compensation mechanism in the kidney cortex, which helps counteract kidney problems [59]. As a result, the increased Na/K-ATPase activity from *T. confluens* venom could be a way for the body to compensate for kidney issues caused by scorpion venom. The notable enzymatic activity seen at lower venom levels (1–10 μg/mL) but not at higher levels (15–20 μg/mL) may indicate a biphasic response. Elevated concentrations can cause enzyme inhibition, protein clumping, or disruption by other venom components, which reduces the overall activity. Such non-linear effects are common in venom research and have been observed in various scorpion and snake venom enzymes [60,61].

No antiproliferative effect was observed after a 24 h treatment at any concentration. The effect was not dose-dependent, as even the highest concentration failed to reduce the number of viable cells. However, the absence of toxicity in the normal cell line could be of interest, as selective cytotoxicity is crucial in antineoplastic therapy [58]. Similar to other Tityus scorpion venoms that have shown little to no direct cytotoxic effects on mammalian cells in vitro, our data suggest that their primary mechanism of toxicity is neurotoxic rather than cytolytic [4,62].

## 4. Conclusions

The first research on *T. confluens* venom revealed a complex composition with seven protein bands, notably at 30, 15, and 10 kDa. Sixteen main fractions were isolated, identifying toxins that target potassium and sodium channels, antimicrobial peptides, hypotensin, and metalloproteinases. The venom also displayed antigenic similarity to *T. serrulatus*, dose-dependent amylolytic activity, increased ATPase activity, and no cytotoxicity in the tested human cell lines. These proteomic results deepen our comprehension of the venom’s clinical impacts and significance for public health.

## 5. Materials and Methods

### 5.1. Specimen Collection and Storage

Specimens (*n* = 30) of *Tityus confluens* (SisGen A9369E2) were collected at night using an ultraviolet lamp, in the urban area of Campo Grande, Mato Grosso do Sul, Brazil (environmental license no. 86953-1). The specimens were identified by Prof. Dr. Leonardo Sousa Carvalho and a voucher was deposited in the Natural History Collection of the Federal University of Piauí, Floriano, 64800-000, PI, Brazil (CHNUFPI). The scorpions were maintained in plastic boxes with water ad libitum. The food consisted of *Drosophila melanogaster* larvae, offered every 2 weeks.

### 5.2. Venom Extraction

The venom extraction was carried out using electro-stimulation [5]. Metal electrodes impregnated with a saline solution (0.9%) were carefully positioned on the telsonic articulation, and a 15 V block signal was applied using an electro-stimulator. After extraction, the venom was centrifuged at 10,500× *g* for 10 min and at 4 °C. The supernatant was pooled and transferred to a microtube (0.5 mL), lyophilized, and stored at −20 °C until use. After extraction, all animals were kept alive in captivity.

### 5.3. Protein Quantification

The protein concentration was estimated according to the Bradford method [63] using bovine serum albumin as a standard and a microplate reader (Spectramex Plus 384—Molecular Devices^®^, San Jose, CA, USA).

### 5.4. Electrophoretic Profile

A sodium dodecyl sulfate polyacrylamide (SDS-PAGE) analysis was performed in 15% gels according to [64], using 25 μg of crude venom for 150 min at 100 V. The proteins were stained with Coomassie Brilliant Blue R-250. The molecular weights were estimated using standard markers (Precision Plus Protein Standards, Bio-Rad, Hongkong). Tricine-SDS-PAGE was also used to separate venom proteins smaller than 30 kDa [65], over a period of 300 min at 85 V.

### 5.5. High-Performance Liquid Chromatographic (HPLC) Analysis

We resuspended 5 mg of lyophilized *T. confluens* venom in 0.1% trifluoroacetic acid (TFA) and centrifuged it at 10,000× *g* for 10 min at 4 °C. Then, we analyzed and fractionated the supernatant using reversed-phase high-performance liquid chromatography (RP-HPLC) in a Shimadzu Prominence binary system (Shimadzu, Kyoto, Japan) with a C18 analytical column (Supelco, 250 × 4.6 mm, 10 μm). We used UV detection (SPDM 20A, Shimadzu, λ = 214 nm) and achieved separation with an optimized linear gradient of 0–60% solvent B (90% acetonitrile, containing 0.1% TFA) over A (0.1% TFA) for 60 min at a constant flow rate of 1 mL/min.

### 5.6. Mass Spectrometry Analyses

The manually collected fractions (50 μL aliquots) underwent in-solution digestion using the following protocol [66]: (1) 3 μL of DTT (100 mM dithiothreitol) was added for 30 min at 60 °C; (2) 4 μL of iodoacetamide (200 mM) was added for another 30 min at room temperature, protected from light; (3) the sample was incubated for at least 16 h at room temperature with 10 μL of trypsin (40 ng/μL in 100 mM ammonium bicarbonate). The reaction was stopped by adding 50% acetonitrile/5% trifluoroacetic acid.

An analysis of the samples was performed using liquid chromatography–mass spectrometry on an ESI-IT-TOF instrument paired with a UPLC 20A Prominence instrument (Shimadzu, Kyoto, Japan). We loaded 15 μL aliquots onto a C18 column (Kinetex C18, 5 μm; 50 × 2.1 mm) and separated them using a binary gradient of solvents: (A) water with acid (990:10) and (B) acetonitrile (ACN) with water and acid (900:99:1). An elution gradient from 0 to 40% B was applied over 80 min at a steady flow rate of 0.2 mL/min after a 5 min initial isocratic run. The eluates were monitored using a Shimadzu SPD-M20A PDA detector before injection into the mass spectrometer.

For the interface, we maintained a voltage of 4.5 kV and a temperature of 275 °C. The detector ran at 1.95 kV and the argon collision-induced fragmentation was set to an ‘energy’ value of 55. We collected the MS spectra in positive mode with a mass range of 350–1400 *m*/*z*, and the MS/MS spectra were collected in the 50–1950 *m*/*z* range.

### 5.7. Data Analysis

Our data processed using the following parameters: the error mass was set to 0.1 Da; methionine oxidation and cysteine carbamidomethylation were used as variable and fixed modifications, respectively; trypsin was the selected cleaving enzyme; the maximum allowed number of missed cleavages was 3, with a maximum of 3 variable PTMs per peptide and non-specific cleavage; the false discovery rate was adjusted to 0.5% or less; only proteins with a score of 20 or higher, containing at least one unique peptide, were considered in this work. The data were analyzed against the Uniprot protein databases using *Tityus* taxid 6886 as the reference. The −10 logP method is a statistical measure used to indicate confidence in peptide or protein identification; the higher the value, the lower the chance of error and the greater the confidence in the identification. The coverage% or protein coverage percentage is the proportion of the protein’s total amino acid sequence that is identified through the peptides detected during the mass spectrometry analysis. Unique peptides refer to peptides found only in that specific protein, while peptides represent the total number of peptides assigned to the protein.

Chromatographic data processing, using the peak area and default LCM Solution 1.24 software parameters, served as the basis for quantitative analyses of the identified proteins. For proteins that co-eluted at the same retention time (meaning they belonged to the same fraction), the number of individual unique peptides was normalized to determine the individual protein’s percentage content in the chromatographic peak, following Equation (1) [67].

Relative protein quantity determination: Rp = ((100 × Af/At) × PUp)/TUp;(1)

Rp = relative protein quantity;
Af = chromatographic peak area (fraction area, UV chromatogram);
At = total chromatographic Area (total UV chromatogram integration);
PUp = protein Unique peptides (integer number of);
TUp = total Unique peptides (integer number of).

### 5.8. Western Blotting

The venom samples (15 µg) separated by 12% SDS-PAGE were electrotransferred at 15 V for 90 min onto nitrocellulose membranes. The membranes were blocked with TBS milk overnight at 4 °C. The membrane was incubated with 1:1000 anti-scorpion serum (batch 190215, Instituto Butantan) for 2 h at room temperature. After washing the blots with Tris-HCl buffer (Tris 10 mM, NaCl 150 mM, pH 7.5) containing 0.1% Tween 20, the membranes were exposed to 1:10,000 peroxidase-labelled anti-horse IgG (Sigma, Clayton, Victoria) for 2 h at room temperature. After washing off unbound secondary antibodies, the immunoreactive bands were visualized using diaminobenzidine (Sigma, Clayton, VIC, USA) and H_2_O_2_. The anti-scorpion serum is derived from the plasma of horses that have been hyperimmunized using venom from *T. serrulatus* scorpions. It is provided in a vial with 5 mL of a purified F(ab′)_2_ fragment of specific immunoglobulins. These immunoglobulins can neutralize at least 1.0 mg of *T*. *serrulatus* venom per milliliter, as shown in mouse seroneutralization tests.

### 5.9. Amylolytic Activity

The amylolytic activity was determined using the MILLER method [68], with adaptations. The 1% (*w*/*v*) soluble starch was prepared in phosphate buffer (6.7 mM NaCl, pH 6.9). The reaction was initiated by adding crude venom (1, 10 or 20 μg) to 15 μL of starch solution. The mixture was incubated at 25 °C for 5 or 10 min. Subsequently, 100 μL of DNS (dinitrosalicylic acid) was added to the test tube, which was boiled for 5 min. The optical density was promptly measured at 540 nm (Spectramex Plus 384—Molecular Devices^®^). One unit of enzyme activity was defined as the amount of enzyme that released 1 µmol of product per minute under standard assay conditions. The results are expressed per milligram of protein (U/mg).

### 5.10. Effect of the Venom on the Total ATPase Activity

The total ATPase activity was determined according to the method previously described using p-nitrophenyl phosphate (pNPP) as the substrate [5]. The ATPase was prepared from rat kidney homogenates’ outer medulla. The kidneys were macerated in storage buffer (12.9 mM imidazole; 0.625 mM EDTA; 250 mM sucrose, titrated to pH 7.4 with HCl). The homogenates were centrifuged at 10,000× *g* for 35 min at 4 °C, and the supernatant was used for determination of the total ATPase activity.

The hydrolysis of pNPP was assessed at 37 °C by monitoring the release of p-nitrophenolate ions (410 nm, pH 7.5, 13,160 M/cm) using a microplate reader (SpectraMax Plus 384, Molecular Devices^®^). The standard conditions were 1 mM EDTA, 50 mM KCl, 3 mM MgCl_2_, 10 mM p-nitrophenyl phosphate, and 30 mM Tris-HCl (pH 7.4). After, different concentrations of venom (1, 5, 10, 15, and 20 μg) were added to the medium. The reaction was initiated by adding 40 μg of homogenate and incubating the solution for 30 min. Controls without enzymes were included in each experiment to quantify the non-enzymatic hydrolysis of the substrate. The initial velocities were constant for at least 15 min, provided that less than 5% of the substrate was hydrolyzed. The results are expressed as nanomoles of p-nitrophenol released per minute and milligram of protein (nmol/min/mg).

### 5.11. Cytotoxicity Assay

The cytotoxicity was assessed using the sulforhodamine B (SRB) colorimetric method [69]. Three cell lines were used—renal carcinoma (CVCL_1051), invasive breast carcinoma (CVCL_0031), and non-tumoral fibroblasts (CVCL_0594). The cells (5 × 10^3^) were plated in 96-well plates and incubated for 24 h. Then, the cells were treated with concentrations of venom (0.1, 1, 10, and 100 μg/mL) and incubated for 48 h. After incubation, the cells were fixed with 20% (*w*/*v*) trichloroacetic acid and stained with 0.4% (*w*/*v*) SRB. The excess dye was removed by washing the cells repeatedly with 1% (*v*/*v*) acetic acid. The protein-bound dye was solubilized with 10 mM Trizma base solution (pH 10.5) for optical density (OD) determination at 540 nm using a microplate reader. Doxorubicin hydrochloride (2 mg/mL) was used as a positive control.

### 5.12. Statistical Analysis

The results are expressed as the mean ± standard deviation (SD) of triplicates. All statistical analyses were conducted using GraphPad Prism software version 5. Multiple comparisons were performed using a one-way analysis of variance (ANOVA), followed by Tukey’s post hoc test. The significance was set at *p* < 0.05.

## Figures and Tables

**Table 1:** Proteins identified in the venom of *Tityus confluens*. The proteins were identified using the Tityus taxid from the NCBI database.

Peak	Access Number	Toxin	Coverage%	LogP	Species	Molecular Mass	Peptides	Unique
5	KBX2_TITTR	Potassium channel toxin TtrKIK	44	140.35	*Tityus trivittatus*	10,067	9	9
6	KBX2_TITTR	Potassium channel toxin TtrKIK	43	162.13	*Tityus trivittatus*	10,067	13	7
6	KA121_TITSE	Potassium channel toxin alpha-KTx 12.1	52	156.37	*Tityus serrulatus*	7083	12	7
6	KBX2_TITCO	Potassium channel toxin TcoKIK	72	120.04	*Tityus costatus*	5284	7	1
7	KBX2_TITTR	Potassium channel toxin TtrKIK	44	129.11	*Tityus trivittatus*	10,067	7	7
8	KBX2_TITTR	Potassium channel toxin TtrKIK	43	175.67	*Tityus trivittatus*	10,067	15	8
9	KBX2_TITTR	Potassium channel toxin TtrKIK	45	161.54	*Tityus trivittatus*	10,067	13	13
9	SCX1_TITTR	Beta-mammal Tt1g	44	116.13	*Tityus trivittatus*	9426	7	5
9	A0A218QX30_TITSE	Putative antimicrobial peptide	14	83.26	*Tityus serrulatus*	9496	6	6
10	SCX4A_TITFA	Beta-toxin Tf4a	83	198.46	*Tityus fasciolatus*	6964	21	19
11	SCX1_TITBA	Toxin Tb1	60	132.95	*Tityus bahiensis*	9384	16	12
12	SCX1A_TITFA	Beta-toxin Tf1a	60	189.90	*Tityus fasciolatus*	9426	16	16
14	KBX1_TITCO	Scorpine-like peptide Tco 41.46-2	36	155.09	*Tityus costatus*	9776	13	13
15	A0AA49K9M8_9SCOR	Hypotensin	47	117.50	*Tityus melici*	8033	5	2
16	VMPA1_TITTR	Venom metalloproteinase	14	130.70	*Tityus trivittatus*	43,062	9	4
16	A0AA49K9T0_9SCOR	Venom metalloproteinase	14	140.41	*Tityus melici*	44,596	7	1

## Data Availability

The original contributions presented in this study are included in the article. Further inquiries can be directed to the corresponding author.

## References

[1] Sinan Sistema de Informação de Agravos de Notificação. https://datasus.saude.gov.br/acesso-a-informacao/doencas-e-agravos-de-notificacao-de-2007-em-diante-sinan/.

[2] Guerra-Duarte C., Saavedra-Langer R., Matavel A., Oliveira-Mendes B.B.R., Chavez-Olortegui C., Bittencourt Paiva A.L. (2023). Scorpion Envenomation in Brazil: Current Scenario and Perspectives for Containing an Increasing Health Problem. PLoS Negl. Trop. Dis..

[3] Pucca M.B., Cavalcante J.S., Jati S.R., Cerni F.A., Ferreira R.S., Arantes E.C. (2025). Scorpions Are Taking over: The Silent and Escalating Public Health Crisis in Brazil. Front. Public Health.

[4] Bernardes-Oliveira E., Farias K.J.S., Gomes D.L., de Araújo J.M.G., Da Silva W.D., Rocha H.A.O., Donadi E.A., Fernandes-Pedrosa M.D.F., Crispim J.C.D.O. (2019). Tityus Serrulatus Scorpion Venom Induces Apoptosis in Cervical Cancer Cell Lines. Evid.-Based Complement. Altern. Med..

[5] Covali-Pontes H.R., Lima Fernandes M.M., Corrêa de Lima L., Rodrigues Macedo M.L., Giannesi G.C., Bastos de Oliveira M.A., Teixeira Ferreira A.M., Farias Frihling B.E., Migliolo L., Pereira dos Santos N.G. (2025). *Tityus paraguayensis*, a Scorpion from the Brazilian Cerrado: First Assessment of Venom and Hemolymph Composition and Biological Activity. Toxicon.

[6] Laraba-Djebari F., Sonia Adi-Bessalem S., Hammoudi-Triki D. (2015). Scorpion Venoms: Pathogenesis and Biotherapies. Scorpion Venoms.

[7] Santibáñez-López C.E., Francke O.F., Ureta C., Possani L.D. (2015). Scorpions from Mexico: From species diversity to venom complexity. Toxins.

[8] Gomes A.C.M., Campos G.P., Rodrigues R.R., Parrela A.F.B., Rodrigues B.S.S.L., Melo-Braga M.N., Junior A.N.R., Siqueira-Batista R. (2022). Escorpiões do Gênero *Tityus* no Brasil: Biologia, Bioquímica da Peçonha e Fisiopatologia do Escorpionismo. Sci. Vitae.

[9] Goldoni P.A.M., Iniesta L.F.M., Marques-da-Silva E., Brescovit A.D. (2025). Adding a Puzzle Piece to the Scorpion Distribution: Expanding the Records of *Tityus (Tityus) confluens* Borelli, 1899 (Scorpiones, Buthidae) in Southern Brazil. Bol. Mus. Para. Emílio Goeldi–Ciências Nat..

[10] Rein J.O. The Scorpion Files. https://www.ntnu.no/ub/scorpion-files/.

[11] Carvalho L.S., Brescovit A.D., Souza C.A.R., Raizer J. (2017). Checklist Dos Escorpiões (Arachnida, Scorpiones) Do Mato Grosso Do Sul, Brasil. Iheringia Ser. Zool..

[12] Lourenço W.R., Cabral B.C., Ramos E.C.B. (2004). Confirmation of *Tityus confluens* Borelli, 1899 (Scorpiones, Buthidae) in Brazil and description of a new subspecies from the State of Mato Grosso do Sul. Bol. Soc. Entomol. Aragonesa.

[13] Ojanguren-Affilastro A., Bizzotto C., Lanari L.C., Remes-Lenicov M., de Roodt A.R. (2019). The Presence of *Tityus Confluens* Borelli in Buenos Aires City and the Expansion of the Distribution of the Medically Important Species of *Tityus* (Scorpiones; Buthidae) in Argentina. Rev. Mus. Argent. Cienc. Nat. Nueva Ser..

[14] Borges A., Rojas de Arias A., Montaño A.M., de Souza C.M.V. (2024). Scorpion Envenoming as an Emerging Public Health Problem in Paraguay, Bolivia, and Midwest Brazil: Involvement of *Tityus Confluens* and the Need for a Panregional Evaluation of Available Antivenoms. Am. J. Trop. Med. Hyg..

[15] Brasil Ministério da Saúde (2024). Guia de Animais Peçonhentos do Brasil.

[16] de Roodt A.R., Lago N.R., Salomón O.D., Laskowicz R.D., Neder de Román L.E., López R.A., Montero T.E., Vega V.d.V. (2009). A New Venomous Scorpion Responsible for Severe Envenomation in Argentina: *Tityus Confluens*. Toxicon.

[17] da Silva Portilho R., Brito I.L., Santos A.N., Moreschi B.P., de Lucena M.N., Otsubo Jaques J.A. (2025). First Evidence of *Tityus Confluens* Borelli, 1899 (Buthidae) Venom Altering Purine Metabolism in Rat Blood Cells. Purinergic Signal.

[18] Âa A., Pessini C., Ãnia T., Takao T., Ãngela E., Cavalheiro C., Vichnewski W., Sampaio S.V., Giglio J.Â.R., Arantes E.C. (2001). A Hyaluronidase from Tityus Serrulatus Scorpion Venom: Isolation, Characterization and Inhibition by flavonoid. Toxicon.

[19] Oukkache N., Chgoury F., Lalaoui M., Cano A.A., Ghalim N. (2013). Comparison between Two Methods of Scorpion Venom Milking in Morocco. J. Venom. Anim. Toxins Incl. Trop. Dis..

[20] Das B., Patra A., Mukherjee A.K. (2020). Correlation of Venom Toxinome Composition of Indian Red Scorpion (*Mesobuthus Tamulus*) with Clinical Manifestations of Scorpion Stings: Failure of Commercial Antivenom to Immune-Recognize the Abundance of Low Molecular Mass Toxins of This Venom. J. Proteome Res..

[21] Rodríguez de la Vega R.C., Possani L.D. (2007). Novel Paradigms on Scorpion Toxins That Affects the Activating Mechanism of Sodium Channels. Toxicon.

[22] Santhosh K.N., Pavana D., Shruthi B.R., Thippeswamy N.B. (2022). Protein Profile of Scorpion Venom from *Hottentotta Rugiscutis* and Its Immunogenic Potential in Inducing Long Term Memory Response. Toxicon.

[23] Valikangas T., Suomi T., Elo L.L. (2018). A comprehensive evaluation of popular proteomics software workflows for label-free proteome quantification and imputation. Brief. Bioinform..

[24] Rauniyar N., Yates J.R. (2014). Isobaric labeling-based relative quantification in shotgun proteomics. J. Proteome Res..

[25] Gstaiger M., Aebersold R. (2009). Applying mass spectrometry–based proteomics to genetics, genomics and network biology. Nat. Rev. Genet..

[26] Calvete J.J. (2013). Snake venomics: From the inventory of toxins to biology. Toxicon.

[27] Cardoso F.C., Lewis R.J. (2018). Sodium Channels and Pain: From Toxins to Therapies. Br. J. Pharmacol..

[28] da Mata É.C.G., Mourão C.B.F., Rangel M., Schwartz E.F. (2017). Antiviral Activity of Animal Venom Peptides and Related Compounds. J. Venom. Anim. Toxins Incl. Trop. Dis..

[29] Neto E.B., de Freitas L.A., Pimenta D.C., Lebrun I., Nencioni A.L.A. (2020). Tb1, a Neurotoxin from *Tityus Bahiensis* Scorpion Venom, Induces Epileptic Seizures by Increasing Glutamate Release. Toxins.

[30] Mantegazza M., Cestèle S. (2005). β-Scorpion Toxin Effects Suggest Electrostatic Interactions in Domain II of Voltage-Dependent Sodium Channels. J. Physiol..

[31] Leipold E., DeBiasi S., Borchardt T., Heinemann S.H. (2012). Scorpion β-Toxin Interference with Na Channel Voltage Sensor Gives Rise to Excitatory and Depressant Modes. J. Gen. Physiol..

[32] Karbat I., Ilan N., Zhang J.Z., Cohen L., Kahn R., Benveniste M., Scheuer T., Catterall W.A., Gordon D., Gurevitz M. (2010). Partial Agonist and Antagonist Activities of a Mutant Scorpion β-Toxin on Sodium Channels. J. Biol. Chem..

[33] Bergeron Z.L., Bingham J.P. (2012). Scorpion Toxins Specific for Potassium (K^+^) Channels: A Historical Overview of Peptide Bioengineering. Toxins.

[34] Bartok A., Panyi G., Varga Z., Gopalakrishnakone P. (2015). Potassium Channel Blocking Peptide Toxins from Scorpion Venom. Scorpion Venoms.

[35] Coronas F.V., De Roodt A.R., Olamendi-Portugal T., Zamudio F.Z., Batista C.V.F., Gómez-Lagunas F., Possani L.D. (2003). Disulfide Bridges and Blockage of Shaker B K+-Channels by Another Butantoxin Peptide Purified from the Argentinean Scorpion *Tityus Trivittatus*. Toxicon.

[36] Holaday S.K., Martin B.M., Fletcher P.L., Krishna N.R. (2000). NMR Solution Structure of Butantoxin. Arch. Biochem. Biophys..

[37] Kalapothakis Y., Miranda K., Aragão M., Larangote D., Braga-Pereira G., Noetzold M., Molina D., Langer R., Conceição I.M., Guerra-Duarte C. (2024). Divergence in Toxin Antigenicity and Venom Enzymes in Tityus Melici, a Medically Important Scorpion, despite Transcriptomic and Phylogenetic Affinities with Problematic Brazilian Species. Int. J. Biol. Macromol..

[38] Novello J.C., Arantes E.C., Varanda W.A., Oliveira B., Giglio J.R., Rgio Marangoni S. (1999). TsTX-IV, a Short Chain Four-Disulfide-Bridged Neurotoxin from Tityus Serrulatus Venom Which Acts on Ca^2+^-Activated K^+^ Channels. Toxicon.

[39] Diego-García E., Batista C.V.F., García-Gómez B.I., Lucas S., Candido D.M., Gómez-Lagunas F., Possani L.D. (2005). The Brazilian Scorpion *Tityus Costatus* Karsch: Genes, Peptides and Function. Toxicon.

[40] de Assis D.R.R., Pimentel P.M., dos Reis P.V.M., Rabelo R.A.N., Vitor R.W.A., Cordeiro M., Felicori L.F., Olórtegui C.D.C., Resende J.M., Teixeira M.M. (2021). *Tityus Serrulatus* (Scorpion): From the Crude Venom to the Construction of Synthetic Peptides and Their Possible Therapeutic Application Against Toxoplasma Gondii Infection. Front. Cell Infect. Microbiol..

[41] Verano-Braga T., Figueiredo-Rezende F., Melo M.N., Lautner R.Q., Gomes E.R.M., Mata-Machado L.T., Murari A., Rocha-Resende C., Elena de Lima M., Guatimosim S. (2010). Structure-Function Studies of *Tityus Serrulatus* Hypotensin-I (TsHpt-I): A New Agonist of B2 Kinin Receptor. Toxicon.

[42] Ettinger K., Cohen G., Momic T., Lazarovici P. (2013). The Effects of a Chactoid Scorpion Venom and Its Purified Toxins on Rat Blood Pressure and Mast Cells Histamine Release. Toxins.

[43] Verano-Braga T., Martins A.L.V., Motta-Santos D., Campagnole-Santos M.J., Santos R.A.S. (2020). ACE2 in the Renin–Angiotensin System. Clin. Sci..

[44] Fletcher P.L., Fletcher M.D., Weninger K., Anderson T.E., Martin B.M. (2010). Vesicle-Associated Membrane Protein (VAMP) Cleavage by a New Metalloprotease from the Brazilian Scorpion *Tityus Serrulatus*. J. Biol. Chem..

[45] Brazón J., Guerrero B., D’Suze G., Sevcik C., Arocha-Piñango C.L. (2014). Fibrin(Ogen)Olytic Enzymes in Scorpion (*Tityus Discrepans*) Venom. Comp. Biochem. Physiol. B Biochem. Mol. Biol..

[46] Ahmadi S., Knerr J.M., Argemi L., Bordon K.C.F., Pucca M.B., Cerni F.A., Arantes E.C., Çalişkan F., Laustsen A.H. (2020). Scorpion Venom: Detriments and Benefits. Biomedicines.

[47] Horta C.C.R., Magalhães B.F., Oliveira-Mendes B.B.R., do Carmo A.O., Duarte C.G., Felicori L.F., Machado-de-Ávila R., Chávez-Olórtegui C., Kalapothakis E. (2014). Identification, Cloning and Functional Characterization of a Hyaluronidase from the Venom of the Brazilian Scorpion *Tityus serrulatus*. PLoS Negl. Trop. Dis..

[48] Almeida D.D., Scortecci K.C., Kobashi L.S., Junqueira-de-Azevedo I.D.L.M., Pimenta D.C., Fernandes-Pedrosa M.F., Kalapothakis E. (2012). Profiling the Resting Venom Gland of the Scorpion *Tityus stigmurus* through a Transcriptomic Survey. BMC Genom..

[49] Almeida F.M., Pimenta A.M., de Figueiredo S.G., Santoro M.M., Martin-Eauclaire M.F., Diniz C.R., de Lima M.E. (2002). Enzymes with Gelatinolytic Activity Can Be Found in *Tityus bahiensis* and *Tityus serrulatus* Venoms. Toxicon.

[50] Dias N.B., de Souza B.M., Cocchi F.K., Chalkidis H.M., Dorce V.A.C., Palma M.S. (2018). Profiling the Short, Linear, Non-Disulfide Bond-Containing Peptidome from the Venom of the Scorpion *Tityus obscurus*. J. Proteom..

[51] Monteiro W.M., Gomes J., Fé N., Silva I.M., Lacerda M., Alencar A., Farias A.S., Val F., Sampaio V.S., Melo G.C. (2019). Perspectives and Recommendations towards Evidence-Based Health Care for Scorpion Sting Envenoming in the Brazilian Amazon: A Comprehensive Review. Toxicon.

[52] Wen F.H., Monteiro W.M., Moura da Silva A.M., Tambourgi D.V., Mendonça da Silva I., Sampaio V.S., dos Santos M.C., Sachett J., Ferreira L.C.L., Kalil J. (2015). Snakebites and Scorpion Stings in the Brazilian Amazon: Identifying Research Priorities for a Largely Neglected Problem. PLoS Negl. Trop. Dis..

[53] Nishikawa A.K., Caricati C.P., Lima M.L., Santos M.C., Kipnis T.L., Eickstedt Z.V., Knysar I., Da M.H., Higashi H.G., Dias Silva W.D. (1994). Antigenic cross-reactivity among the venoms from several species of Brazilian scorpions. Toxicon.

[54] Cid-Uribe J.I., Veytia-Bucheli J.I., Romero-Gutierrez T., Ortiz E., Possani L.D. (2020). Scorpion Venomics: A 2019 Overview. Expert. Rev. Proteom..

[55] Magalhães A.C.M., de Santana C.J.C., Melani R.D., Domont G.B., Castro M.S., Fontes W., Roepstorff P., Júnior O.R.P. (2021). Exploring the Biological Activities and Proteome of Brazilian Scorpion *Rhopalurus Agamemnon* Venom. J. Proteom..

[56] Chen C.C., Lin-Shiau S.Y. (1985). Mode of Inhibitory Action of Melittin on Na^+^-K^+^-ATPase Activity of the Rat Synaptic Membrane. Biochem. Pharmacol..

[57] Perera Córdova W.H., Leitão S.G., Cunha-Filho G., Bosch R.A., Alonso I.P., Pereda-Miranda R., Gervou R., Touza N.A., Quintas L.E.M., Noël F. (2016). Bufadienolides from Parotoid Gland Secretions of Cuban Toad *Peltophryne Fustiger* (Bufonidae): Inhibition of Human Kidney Na^+^/K^+^-ATPase Activity. Toxicon.

[58] De Sousa L.Q., da Conceição Machado K., de Carvalho Oliveira S.F., da Silva Araújo L., dos Santos Monção-Filho E., de Carvalho Melo-Cavalcante A.A., Vieira-Júnior G.M., Ferreira P.M.P. (2017). Bufadienolides from Amphibians: A Promising Source of Anticancer Prototypes for Radical Innovation, Apoptosis Triggering and Na+/K+-ATPase Inhibition. Toxicon.

[59] Linardi A., Rocha E., Silva T.A.A., Miyabara E.H., Franco-Penteado C.F., Cardoso K.C., Boer P.A., Moriscot A.S., Gontijo J.A.R., Joazeiro P.P. (2011). Histological and Functional Renal Alterations Caused by *Bothrops Alternatus* Snake Venom: Expression and Activity of Na^+^/K^+^-ATPase. Biochim. Biophys. Acta Gen. Subj..

[60] Amorim F.G., Longhim H.T., Cologna C.T., Degueldre M., Pauw E.d., Quinton L., Arantes E.C. (2019). Proteome of Fraction from Tityus serrulatus Venom Reveals New Enzymes and Toxins. J. Venom Anim. Toxins Trop. Dis..

[61] Doley R., Kini R.M. (2009). Protein Complexes in Snake Venom. Cell. Mol. Life Sci..

[62] Furtado A.A., Daniele-Silva A., Silva-Júnior A.A., Fernandes-Pedrosa M.F. (2020). Biology, Venom Composition, and Scorpionism Induced by Brazilian Scorpion *Tityus stigmurus* (Thorell, 1876) (Scorpiones: Buthidae): A Mini-Review. Toxicon.

[63] Bradford M.M. (1976). A Rapid and Sensitive Method for the Quantitation Microgram Quantities of Protein Utilizing the Principle of Protein-Dye Binding. Anal. Biochem..

[64] Laemmli U.K. (1970). Cleavage of Structural Proteins during the Assembly of the Head of Bacteriophage T4. Nature.

[65] Schägger H., von Jagow G. (1987). Tricine-Sodium Dodecyl Sulfate-Polyacrylamide Gel Electrophoresis for the Separation of Proteins in the Range from 1 to 100 KDa. Anal. Biochem..

[66] Fusco L.S., Neto E.B., Francisco A.F., Alfonso J., Soares A., Pimenta D.C., Leiva L.C. (2020). Fast Venomic Analysis of *Crotalus Durissus* Terrificus from Northeastern Argentina. Toxicon X.

[67] Beraldo-Neto E., Vigerelli H., Coelho G.R., da Silva D.L., Nencioni A.L.A., Pimenta D.C. (2023). Unraveling and profiling Tityus bahiensis venom: Biochemical analyses of the major toxins. J. Proteom..

[68] Miller G.L. (1959). Use of Dinitrosalicylic Acid Reagent for Determination of Reducing Sugar. Anal. Chem..

[69] Skehan P., Storeng R., Scudiero D., Monks A., Mcmahon J., Vistica D., Warren J.T., Bokesch H., Kenney S., Boyd M.R. (1990). New Colorimetric Cytotoxicity Assay for Anticancer-Drug Screening. J. Natl. Cancer Inst..

